# Suicidal Ideation and Associated Factors among School-Going Adolescents in Thailand

**DOI:** 10.3390/ijerph9020462

**Published:** 2012-01-31

**Authors:** Karl Peltzer, Supa Pengpid

**Affiliations:** 1 HIV/AIDS/STI and TB (HAST), Human Sciences Research Council, Private Bag X41, Pretoria 0001, South Africa; 2 Department of Psychology, University of Limpopo, Turfloop Campus, Private Bag X1106, Sovenga 0727, South Africa; 3 Department of Health System Management and Policy, University of Limpopo, Medunsa Campus, P.O. Box 197, Medunsa 0204, South Africa; Email: supa_pengpid@embanet.com

**Keywords:** suicidal ideation, psychosocial distress, social-environmental factors, substance use, adolescent health, Thailand

## Abstract

The aim of this study was to estimate the prevalence and identify associations between suicidal ideation and indicators of psychosocial distress and social-environmental factors in Thai adolescents. Using data from the Thailand Global School-Based Student Health Survey (GSHS) 2008, we assessed the prevalence of suicidal ideation and its associated factors among adolescents (N = 2,758). Overall the prevalence of suicidal ideation in the past 12 months was 8.8% (9.9% males and 7.7% females). Variables influenced the suicidal ideation in multivariable analysis were sadness (Odds Ratio = OR: 6.03; 95% Confidence Interval = CI (3.00–12.14), lack of parental attachment (OR = 2.26, CI = 1.09–4.67), current alcohol use (OR = 2.32, 1.21–4.44), and ever having had sexual intercourse (OR = 4.16, CI = 3.40–7.68). Psychosocial, health-risk behaviours and lack of protective factors appear to effect suicidal ideation in this youth population.

## 1. Introduction

Among youth (15 to 19 years) suicide is the fourth leading cause of death globally [1] and among 10 to 24 years 6% of all deaths globally in 2004 were attributed to suicide [2]. Suicidal behaviour includes suicidal ideation (ideas/thoughts), plans, attempts and ultimately, death, through a specific action [3]. Suicidal ideation with a plan to attempt suicide is usually much less frequent than having thoughts or contemplation of suicide [4]. Studies among adolescents from low and middle income countries seem to show considerable rates of prevalence of suicidal ideation: 17.8% in China [5], 17.1% in the Philippines [6], in Lebanon 16% [7], and in African countries 19.6% in Uganda, 23.1% in Botswana, 27.9% in Kenya, and 31.9% in Zambia [8]. Local studies in Thailand found among high school students 4.0% suicidal ideation [9], among student youth in Bangkok 6.1% attempted suicide [10], among high school students 4.6% had attempted suicide during the last year [11], and in a study in Chiang Mai, high school students among boys 5.7% and girls 7.4% suicide attempts in the past 12 months [6]. Lotrakul [12] notes that in Thailand suicide rates were found to have increased to a peak of 8.6 per 100,000 (5,290 suicides) in 1999 and then to have decreased to 7.1 per 100,000 in 2003 [12]. The average suicide rate during 1998–2003 was 7.9 per 100,000 with a male to female ratio of 3.4:1. Male suicide reached a peak for those aged 25–29 years (21.9 per 100,000) while female suicide showed less variation with age [12]. Hanging was the most common method used, followed by ingestion of agricultural toxic substances. Suicide was most prevalent in the upper northern region where HIV infection might be related to the high prevalence [12].

Factors associated with risk of adolescent suicidality (suicidal ideation, suicidal behaviour, death by suicide) have been identified as social integration, perceptions of family and peer support, childhood abuse/neglect, and peer victimization [13]. In general, psychiatric, biological, social and environmental factors related to an individual‘s life history are risk factors for suicide [14]. For this study, risk factors have been categorized into three groups: (1) psychosocial; (2) social-environmental; and (3) socio-demographic [15]. Considering these three factors, studies from low and middle income countries found the following factors associated with adolescent suicidality: (1) psychosocial: hopelessness and loneliness [7,8,16,17], psychosocial distress (loneliness, worry) [6,18]; (2) social-environmental: parental and peer protective factors, such as having parents check homework and understand their problems/worries and having students in their school who were kind and helpful most of the time or always, lowered the risk of suicidal ideation [7,17]; school attachment (truancy [16]), physically abused at school (*i.e.*, bullied) [7,19]; substance use (tobacco smoking, having been drunk, illicit drug use) [7,18,20,21,22]; other health risk behaviour such sexual activity (ever sexual intercourse) [21]; Overweight status and sedentary behaviours [18], physical activity [18]; and (3) socio-demographic: being male [14,23], being female [18,23].

The current study is innovative because it expands the present research on adolescent suicidal ideation to an exploration of the association of psychosocial distress and social-environmental factors in a culturally distinct population of Thai adolescents. We hypothesized that similar to previous research, mainly in low and middle income countries, psychosocial distress and social-environmental factors would be associated with increased risk of suicidal ideation.

Therefore, the aim of this study was to obtain the prevalence of, and assess factors between suicidal ideation and indicators of psychosocial distress and social-environmental factors in Thai adolescents.

## 2. Methods

### 2.1. Participants and Procedures

The study involved the secondary analysis of existing data from the 2008 Thailand Global School-Based Health Survey (GSHS). The aim of the GSHS is to collect data from students of age 13 to 15 years. The Thailand GSHS was a school-based survey of students in Grades 7, 8, 9, and 10. A two-stage cluster sample design was used to collect data to represent all students in Grades 7, 8, 9, and 10 in the country. At the first stage of sampling, schools were selected with probability proportional to their reported enrolment size. In the second stage, classes in the selected schools were randomly selected and all students in selected classes were eligible to participate irrespective of their actual ages. The school response rate was 100%, the student response rate was 93%, and the overall response rate (in this case the student response rate) was 93%. Students self-completed the questionnaires to record their responses to each question on a computer scan able answer sheet. A total of 2,767 students participated in the Thailand GSHS [24]. The GSHS 10 core questionnaire modules address the leading causes of morbidity and mortality among children and adults worldwide: tobacco, alcohol and other drug use; dietary behaviors; hygiene; mental health; physical activity; sexual behaviors that contribute to HIV infection, other sexually transmitted infections, and unintended pregnancy; unintentional injuries and violence; protective factors and respondent demographics [24,25].

### 2.2. Measures

*Suicidal ideation*: “During the past 12 months, did you ever seriously consider attempting suicide?” (response option was 1 = yes and 2 = no, recoded 1 = 0, 2 = 1).

#### 2.2.1. Socio-Demographics

Sex, age and school grade was assessed. In addition, a measure of economic status: *Hunger*. A measure of hunger was derived from the question “During the past 30 days, how often did you go hungry because there was not enough food in your home?” (response options were from 1 = never to 5 = always; recoded 1 = never, rarely and 0 = sometimes, most of the time or always).

#### 2.2.2. Psychosocial Factors

*Anxiety or worried*. During the past 12 months, how often have you been so worried about something that you could not sleep at night? (Response options were from 1 = never to 5 = always) (Recoded 1 = most of the time or always and 0 = never, rarely or sometimes).

*Sadness*. During the past 12 months, did you ever feel so sad or hopeless almost every day for 2 weeks or more in a row that you stopped doing your usual activities? (Response option 1 = yes and 2 = no) (Recoded 1 = 1, 2 = 0).

#### 2.2.3. Social-Environmental Factors

*Lack of parental attachment*: Parental attachment was assessed with three items. (1) Parental or guardian supervision: “During the past 30 days, how often did your parents or guardians check to see if your homework was done”? (2) Parental or guardian connectedness: “During the past 30 days, how often did your parents or guardians understand your problems or worries?” and (3) Parental or guardian bonding “During the past 30 days, how often did your parents or guardians really know what you were doing with your free time?” Response options to these questions were from 1 = never to 5 = always, recoded 1 = never or rarely and 0 = sometimes to always. The three parental attachment items were added together to form a “lack of parental attachment index”, the Cronbach alpha of this index was 0.67 in this sample. This index was further recoded into 0 = 0, 1–2 = 1 and 3 = 2, indicating low, medium and high lack of parental attachment.

*Lack of peer support*: at school was assessed with the question “During the past 30 days, how often were most of the students in your school kind and helpful?” Response options to this question was from 1 = never to 5 = always, recoded 1 = never or rarely and 0 = sometimes to always.

*Truancy*: During the past 30 days, on how many days did you miss classes or school without permission? (Response options were from 1 = 0 times to 5 = 10 or more times) (recoded 1 = 1 or 2 to 10 or more times, and 0 = 0 times)

*Exposure to bullying*: To assess exposure to bullying behaviour, students were prompted with the following: “Bullying occurs when a student or group of students say or do bad and unpleasant things to another student. It is also bullying when a student is teased a lot in an unpleasant way or when a student is forced to withdraw from certain activities on purpose. It is not bullying when two students of about the same strength or power argue or fight or when teasing is done in a friendly and hilarious way.” Students were then asked the following question: “During the past 30 days, on how many days were you bullied?” Response options ranged from 1 = 0 days to 7 = all 30 days. Those reporting one or more days were considered to have been bullied. 

*Substance use variables*: Tobacco use: (i) smoking cigarettes (current smoking) and (ii) use of any other form of tobacco (current other tobacco use) in the past 30 days. Tobacco use was assessed with the question, “During the past 30 days, on how many days did you smoke cigarettes (use other tobacco products?” Response options included 1 = 0 days to 7 = all 30 days. Current tobacco use was defined as smoking cigarettes and/or use of other tobacco products in the past 30 days.

Alcohol use was assessed with the question, “During the past 30 days, on how many days did you have at least one drink containing alcohol?” Response options included 1 = 0 days to 7 = all 30 days. Drug use: ‘During your life, how many times have you used drugs, such as methamphetamines (Yaba), ecstasy, 4 × 100, or marijuana? Response options ranged from 1 = 0 times to 4 = 10 or more times. 

*Physical inactivity*: Leisure time physical activity was assessed by asking participants: “During the past 7 days, on how many days were you physically active for a total of at least 60 minutes per day?” and “During a typical or usual week, on how many days are you physically active for a total of at least 60 minutes per day?” Physical activity was defined as any activity that increases heart rate and makes one get out of breath some of the time. Physical activity can be done in sports, playing with friends, or walking to school. Some examples of physical activity are running, fast walking, biking, dancing, and football. Physical education or gym classes were not supposed to be included. According to the scoring protocol of the PACE+Adolescent Physical Activity Measure and existing guidelines, physical inactivity was defined as obtaining less than 60 min of physical activity per day on at least five days per week. For analysis, the number of active days “during the past week” and the number of active days “during a typical week” were averaged. 

*Obesity:* Height and body weight were based on self-reports. Body Mass Index (BMI) was calculated as weight/height^2^ (kg/m^2^). The international age- and gender-specific child BMI cut-points were used to define underweight, overweight and obesity [26]. These cut-points were derived in a large international sample using regression techniques by passing a line through the health-related adult cut-points at 18 years. Youth with BMI values corresponding to an adult BMI of <25.0 kg/m^2^ were classified as normal weight and youth with BMI values corresponding to an adult BMI of ≥25.0 kg/m^2^ were classified as overweight. Thus, in this study obese included those with a BMI corresponding to adult value of ≥30.0 kg/m^2^. The response rate on the BMI was for Thailand 97%.

*Sexual behaviours*: The sexual behaviour item included in this analysis was: ‘How old were you when you had sexual intercourse for the first time?’ Response options ranged from I have never had sexual intercourse to 16 years old or older. Ever had sexual intercourse was recoded to 1 and never to 0.

### 2.3. Data Analysis

Data analysis was performed using STATA software version 10.0 (Stata Corporation, College Station, TX, USA). This software has the advantage of directly including robust standard errors that account for the sampling design, *i.e.*, cluster sampling owing to the sampling of school classes. Data were checked for normality distribution and outliers. Potential multi-collinearity between variables was assessed with variance inflation factors, none of which exceeded the recommended critical value of 4.0 [27]. Associations between suicidal ideation and socio-demographic, substance use, psychosocial distress variables and protective factors were evaluated calculating odds ratios (OR). Logistic regression was used for evaluation of the effect of explanatory variables for suicide ideation in the past 12 months (binary dependent variables). All variables statistically significant at the *P* < 0.05 level in bivariate analyses were included in the multivariable models. In the analysis, weighted percentages are reported. The reported sample size refers to the sample that was asked the target question. The two-sided 95% confidence intervals are reported. The P values less or equal to 5% is used to indicate statistical significance. Both the reported 95% confidence intervals and the P value are adjusted for the multi-stage stratified cluster sample design of the study.

## 3. Results

Table 1 gives the sample characteristics of 2,758 participants, mainly between 12 to 15 years old and 53.2% females and 46.8% males. Overall the prevalence of suicidal ideation was 8.8% (9.9% males and 7.7% females) in the past 12 months. Regarding psychosocial distress, 16.5% reported sadness and 6.5% anxiety. In terms of social-environmental factors, 37.3% reported high lack of parental or guardian attachment, 58.3% lack of peer support, 17.1% truancy, 27.8% being bullied, 9.5% tobacco and 14.8% alcohol use in the past month, 6.0% ever illicit drug use, 4.4% had been obese and 6.1% ever had sexual intercourse. Substance use, sexual behaviour, being bullied and truancy variables were all higher among males than females (see Table 1).

**Table 1:** Sample characteristics among adolescents in Thailand, 2008, N = 2,758.

	Total	Males	Females
	N (%)	N (%)	N (%)
Suicidal ideation	235 (8.8)	129 (9.9)	104 (7.7)
**Socio-demographics**			
Age (years)			
≤12 (of which 11 years or less = 0.5%)	466 (16.9)	201 (15.6)	265 (18.2)
13	840 (29.5)	407 (30.9)	433 (28.1)
14	870 (28.7)	443 (30.3)	427 (27.2)
≥15 (of which 16 years or more = 3.7%)	582 (24.9)	313 (23.2)	269 (26.5)
Education			
Grade 7	783 (29.5)	385 (30.8)	398 (28.2)
Grade 8	847 (28.6)	400 (29.4)	447 (27.7)
Grade 9	879 (28.1)	442 (28.4)	437 (27.9)
Grade 10	227 (13.8)	129 (11.4)	98 (16.2)
Gender			
Female	1,394 (53.2)		
Male	1,364 (46.8)		
Hunger	94 (3.4)	63 (4.7)	31 (2.1)
**Psychosocial distress**			
Sad	446 (16.6)	233 (18.2)	212 (15.1)
Anxiety	186 (6.5)	91 (6.6)	95 (6.5)
**Social-environmental factors**			
Lack of parental/guardian attachment			
Low (0)	447 (16.5)	169 (12.9)	278 (19.8)
Medium (1–2)	1,240 (46.2)	607 (45.4)	633 (46.9)
High (3)	1,000 (37.3)	547 (41.7)	453 (33.3)
Lack of peer support	1,583 (58.3)	872 (65.6)	711 (51.5)
Truancy	467 (17.1)	317 (24.0)	150 (10.6)
Being bullied	679 (27.8)	383 (32.9)	296 (23.2)
Tobacco use in the past month	253 (9.5)	217 (17.1)	36 (2.6)
Alcohol use in the past month	368 (14.8)	247 (21.2)	121 (9.3)
Ever illicit drug use	167 (6.0)	147 (11.1)	20 (1.3)
Physically inactive	2,073 (76.3)	914 (67.5)	1,159 (84.6)
Obesity	118 (4.4)	67 (5.0)	51 (3.9)
Ever had sexual intercourse	145 (6.1)	87 (7.5)	58 (4.9)

### 3.1. Association Between Suicidal Ideation and Factors Assessed

In bivariate analysis hunger, sadness, anxiety, lack of parental attachment, lack of peer support, truancy, being bullied, substance use and ever having had sexual intercourse were associated with suicidal ideation, while in multivariable logistic regression current alcohol use, sexual activity (ever sex), psychosocial distress (sadness) and lack of parental or guardian attachment were associated with suicidal ideation (see Table 2).

**Table 2:** Logistic regression analysis of the likelihood of having a suicidal ideation compared not having suicidal ideation among adolescents in Thailand, 2008.

	UOR (95% CI)	SE	AOR (95% CI)	SE
**Socio-demographics**				
Age (years)				
≤12	1.00		1.00	
13	0.95 (0.56–1.60)	0.23	0.62 (0.22–1.77)	0.30
14	0.84 (0.49–1.45)	0.22	1.00 (0.29–3.40)	0.57
≥15	0.85 (0.47–1.52)	0.23	0.73 (0.21–2.57)	0.43
Education				
Grade 7	1.00		1.00	
Grade 8	1.05 (0.77–1.44)	0.16	1.35 (0.56–3.26)	0.56
Grade 9	0.67 (0.42–1.07 )	0.15	0.58 (0.26–1.33)	0.22
Grade 10	1.02 (0.57–1.85)	0.28	0.84 (0.34–2.11)	0.36
Gender				
Female	1.00		1.00	
Male	1.32 (1.57–4.49)	0.26	0.93 (0.48–1.80)	0.29
Hunger	2.66 (1.57–4.49) ***	0.66	0.89 (0.24–3.33)	0.55
**Psychosocial distress**				
Sad	7.29 (4.72–11.24) ***	1.48	6.03 (3.00–12.14) ***	1.98
Anxiety	4.11 (2.69–6.26) ***	0.81	1.42 (0.75–2.69)	0.43
**Social-environmental factors**				
Lack of parental/guardian attachment				
Low (0)	1.00		1.00	
Medium (1–2)	2.46 (1.32–4.57) **	0.72	1.72 (0.76–3.93)	0.67
High (3)	4.23 (2.48–7.22) ***	1.06	2.26 (1.09–4.67) *	0.80
Lack of peer support	1.69 (1.28–2.22) ***	0.22	1.58 (0.82–3.03)	0.48
Truancy	2.62 (1.83–3.73) ***	0.44	0.78 (0.36–1.68)	0.28
Being bullied	2.89 (1.88–4.43) ***	0.58	1.64 (0.76–1.69)	0.59
Tobacco use in past month	3.24 (1.91–5.52) ***	0.81	0.82 (0.25–2.64)	0.45
Alcohol use in past month	3.36 (2.28–4.96) ***	0.61	2.32 (1.21–4.44) *	0.71
Ever illicit drugs	3.79 (2.90–4.97) ***	0.48	1.56 (0.49–4.93)	0.84
Physically inactive	1.28 (0.93–1.77)	0.19	---	
Obesity	0.99 (0.43–2.26)	0.38	---	
Ever had sexual intercourse	4.16 (2.59–6.68) ***	0.92	3.40 (1.51–7.68) **	1.30

*** P < 0.001; ** *P* < 0.01; * *P* < 0.05

## 4. Discussion

Studies of adolescent suicidal ideation are important because thoughts about suicide are associated with the intend to commit suicide and actual attempted suicides [4,21,28]. This study found an overall prevalence of suicidal ideation of 8.8% (9.9% males and 7.7% females) in the past 12 months among school-going adolescents in Thailand. This rate seems to be higher than in previous local studies among adolescents (4.6%) [9], 4% in Thailand [10] and countries studies in the region (Myanmar 0.7%, Indonesia 4.0%) [29], similar in Sri Lanka (9.9%) [29], and lower than in a number of other low and middle income countries from 16% in Lebanon, 17.1% in China to 31.9% in Zambia [5,7,8,24,28,30]. The low rate of suicidal ideation in Thailand may be due to Buddhism, about 95% of the population are Buddhist and one of the five ‘immoral actions’ of Buddhism requires a lay-Buddhist to train himself or herself to avoid destroying life [31]. Religious affiliation and practice among Thai people may play an important role in preventing them from suicidal behaviour when they encounter hopeless feelings in their lives [32]. 

The primary purpose of the current study was to identify the association of suicidal ideation and psychosocial distress and social-environmental factors in a distinct sample of Thai adolescents. Previous research across age groups has identified psychiatric disorders as important risk factors for suicidal behaviour [33]. Psychosocial distress such as sadness and hopelessness may increase to unbearable levels leading to suicidal ideation, attempts, or actual suicide [34]. Consistent with previous research [7,8,16,22,35], we found that having feelings of sadness was the overall strongest predictor of suicidal ideation in this study. 

Further, this study found that the lack of parental attachment was associated with suicidal ideation. Other studies found similar results even to the extent of multiple protective factors [17]. Utilizing protective factors, school-based targeted education programmes for parents of adolescents have shown evidence that they can improve protective factors [17]. Substance use has been identified as a correlate of suicidal behavior in low and middle income countries [7,18,22,23]. Given such previous findings, alcohol use was surprisingly only significantly associated with suicidal ideation, as also found in studies among high school students in Thailand [9,36].

In terms of other health risk behaviours, this study found that sexual activity (ever sex) was associated with suicidal ideation, which was also found among Chinese [19], Venezuelan [37] and Estonian school children [38]. This factor may be indirectly associated with suicidal ideation. Adolescent sexual activity, within or outside of marriage, can lead to negative reproductive health outcomes. Unprotected sexual activity can expose young women to the risks of unintended pregnancy, unwanted childbearing and abortion, as well as HIV and other sexually transmitted infections [39,40]. Previous analyses of motives behind a young person’s decision to become sexually active have shown that external pressure to have sex plays a significant role [41], which can accompany psychological distress [38]. Previous studies found that stressful life events are often followed by impulsive suicidal behaviours [14,33,42,43]. Some authors [44] have described “clustering” of unhealthy or risk behaviours including alcohol use, sexual intercourse and suicidal ideation.

However, other health risk behaviours (bully victimization, tobacco use, illicit drug use, physical inactivity, obesity and truancy) were in this study not associated with suicidal ideation, unlike in some other studies [7,16,18,20,23]. The study did not find any gender differences in terms of suicidal ideation, which was also found in Lebanon [7]. Most studies among adolescents in low and middle income countries show either higher rate among boys [15,23] or among girls [16,17,18,19]. In addition, suicidal ideation did not increase with adolescent age, which is conform with some other studies among adolescents [7,17], while in one study among Chinese adolescents suicidal ideation did increase with age [17,28].

## 5. Study Limitation

This study had several limitations. Firstly, the GSHS only enrolls adolescents who are in school. School-going adolescents may not be representative of all adolescents in a country as the occurrence of illicit drug use may differ between the two groups. Also we did not assess regional and urban-rural differences in suicidal ideation. As the questionnaire was self-completed, it is possible that some study participants may have miss reported either intentionally or inadvertently on any of the questions asked. Intentional miss reporting was probably minimized by the fact that study participants completed the questionnaires anonymously. The questionnaire used in this study measured different concepts such as psychosocial distress variables with single items, which are quite limited in their use as quantitative indices. Another limitation is that suicide attempts were not assessed in this study. A number of risk factors associated with suicidal ideation and behaviour found in other studies such as life events [4,14], childhood violence [45], childhood abuse/neglect [13], and academic performance [28] were not assessed and should be assessed in future studies. Furthermore, this study was based on data collected in a cross-sectional survey. We cannot, therefore, ascribe causality to any of the associated factors in the study. Longitudinal studies of suicidal behaviour are necessary to understand the etiological pathways from protective factors to suicide prevention in adolescents [17].

## 6. Conclusion

A moderate level of suicidal ideation was found in this sample of school-going adolescents in Thailand. Certain risk factors associated with suicidal behaviour were identified which can be taken into consideration in the development and implementation of interventions to prevent suicide.
